# Science Diplomacy in Emerging Economies: A Phenomenological Analysis of the Colombian Case

**DOI:** 10.3389/frma.2021.636538

**Published:** 2021-04-30

**Authors:** Luisa Fernanda Echeverría King, Diana Alejandra González, Ernesto Andrade-Sastoque

**Affiliations:** ^1^Department of Research and Diagnostic Methods in Education, Universidad de Murcia, Murcia, Spain; ^2^International Cooperation and Visibility, Universidad de La Sabana, Chía, Colombia; ^3^STePS Research Group, University of Twente, Enschede, Netherlands

**Keywords:** science diplomacy, emerging economies, international cooperation, research policy, policy mix, governance

## Abstract

Little has been investigated about Science Diplomacy (SD) in emerging economies, more specifically on governance schemes useful for organizing intersecting actors, practices, conceptions and suggestions of the future in foreign affairs and Science, Technology and Innovation (STI) in public administration sectors. This paper contributes to a better understanding of the “texture and nature” of SD initiatives in emerging economies through the eyes of relevant actors involved or reflecting about them in Colombia. The aim of this paper is to propose a general governance scheme for SD in emerging economies and its potential instrumentation for a policy-mix. In Colombia, SD initiatives are very fragmented, and are not part of the priorities of the Colombian state, however the increasing interest of an embryonic practitioner and scholar community working in the topic make necessary this work. A phenomenological perspective combined with a single case study research methodology is used to gain a very accurate description of the state of the situation of SD in Colombia. Policy document review and semi-structured interviews were conducted with 18 relevant actors to understand the conceptions, practices, and suggestions for the future of SD in Colombia. The study results show that SD actors in Colombia are scattered, practices strongly related to traditional cooperation diplomatic activities and the need to give a function to SD for capacity building, better global intermediation and the development of new knowledge, in particular promoting SD abilities in the scientific community. In addition, data expresses the need to cultivate a multi-stakeholder working group for such a purpose. The study reflects on the need of a policy mix for SD in emerging economies. It proposes a general governance scheme for it, a potential instrumentation founded on research participant future suggestions, and a set of practical recommendations and policy implications. Conclusions and further research questions are set, pointing out the importance of including non-conventional diplomacy actors and knowledge, and the need to inquire rationales behind possible SD policy mixes in the southern world.

## Introduction

The interaction between science and diplomacy is becoming more and more necessary for governments to tackle global challenges. Such interaction has been defined recently as a process and a set of practices at the intersection of these two domains, what is called Science diplomacy (SD) (S4D4C, 2019). SD initiatives are mechanisms to promote and strengthen science, technology and innovation (STI) activities at the national level. In emerging economies, SD has been the basis for the generation of research centers, bilateral projects, mobility programs for scientists, and capacity building actions in STI. In Latin America, the approaches to SD are diverse. Panama, for example, developed in 2018 a SD policy with the leadership of the Ministry of Foreign Affairs. Other countries in the region such as Brazil, Mexico, and Cuba have also implemented their own strategies.

Most of the literature that delves into the concept of SD in emerging economies has a focus on specific instruments to promote STI cooperation (Hornsby and Parshotam, 2018), case studies focused on areas and dynamics of collaboration (Frech et al., 2018), capacity-building recommendations for individuals involved in SD (Krasnyak, 2020), and analysis of bi-regional agreements (Cherry and Du Toit, 2018). In Colombia SD initiatives are scattered, there is no state's strategy for it, and there exists a lack of coordination between relevant actors involved in such initiatives. On the one hand, inside the Ministry of Foreign Affairs there is an absence of SD discourse, and on the other hand, in the Science, Technology and Innovation Ministry there is lack of long-term initiatives of SD. However, due to (i) the peace agreement signed in 2016; (ii) the creation of the STI Ministry; (iii) the increasing interest of the government in promoting innovation as the country's engine for sustainable development; (iv) the coronavirus pandemic's public health crisis, among other reasons; there is a valuable opportunity for Science Diplomacy, especially since the efforts and coordination actions between the countries and the scientific advice required in sustainable development challenges have been essential to face the current global crisis (Vargas Solorzano, 2020). Bluntly put, there is a unique opportunity for setting SD in the country's policy agenda.

In order to analyze the existing literature, four categories were determined. According to the research objectives of the present study: (i) actors, (ii) practices, (iii) conceptions, and (iv) suggestions for the future of SD in emerging economies. There are a number of references in SD in emerging economies literature that point out to non-traditional actors of diplomacy (Pantović and Michelini, 2018, Hornsby and Parshotam, 2018, Ezekiel, 2020). Practices mentioned mostly draw to the broader dynamics of governmental support to STI collaboration (Patman and Davis, 2017; Thompson, 2018; Ezekiel, 2020) however the purpose of such practices refers to: promotion, influence and access (Flink and Schreiterer, 2010). When reviewing conceptions category, the literature on SD in emerging economies is mainly focused on *access*, this is, the development of capacities and exchange of resources in order to be part of the global scene of STI (Hornsby and Parshotam, 2018; Thompson, 2018). Regarding suggestions category for SD in emerging economies we found three main issues, (i) the need for scientists to assume leading roles in political debates, advising policy makers with scientific results and data (Patman and Davis, 2017); (ii) Foreign policy requires the integration of scientific evidence in their work to be able to support the implementation of SD initiatives (Ramírez-Cabrales and Rueda Forero, 2020); (iii) the need to identify spaces for dialogue between academics, researchers, and decision makers, in order to articulate projects to solve global issues and to address foreign policy priorities (Pantović and Michelini, 2018); as well as (iv) the need of new models of SD, including diverse actors to generate exchange and new knowledge (Thompson, 2018).

There is an important scholarship on SD in emerging economies that has been developed in Sub-Saharan Africa, Latin America, Russia, the Balkans, among others. However, the reviewed cases on Russia, India, and Brazil, are key. The Russian case shows how the Cold War, and the accession to Crimea resulted affecting the STI dynamics of the country (Ibragimova and Barabanov, 2018) and how the country nowadays, recognizes the importance of SD for tackling these issues (Krasnyak, 2020). For example, most recent actions in the country have been focused on the attraction and retention of Russian researchers (Ibragimova and Barabanov, 2018) which can restore the international image of Russian research system (Ruffini, 2017). In India, SD policy is focused in the importance of the acquisition, exchange and development of technologies through strategic alliances (Sikka, 2017), as well as the relevance to support other emerging economies through SD initiatives such as scholarships awarded to foreign scientists and experts under the Indian Technical and Economic Cooperation Program (Arunachalam et al., 2017). In the Brazilian case, the strategic approach within the South-South cooperation schemes is paramount. The concept of SD was first adopted and developed by the Ministry of External Affairs (Itamaraty), by creating the National Program on Innovation Diplomacy that is focused on acquiring a new productive-technological profile to allow Brazil to redefine its international positioning and insertion in the global economy (Anunciato and dos Santos, 2020). As it can be seen the evolution of SD in emerging economies is very dependent on the specific historical and socio-economic conditions of each country or region.

Nevertheless, there is not enough developed work about the governance of SD in emerging economies, particularly in Latin American countries. In Latin America, while many of the countries share similar historic and cultural traits, the evolution and integration of SD as a concept within the discourse of national STI and foreign policies is quite varied. There are countries in the region that are still in the very early stages of defining a strategic approach about it and still struggle to gain the necessary momentum to reach a national agreement and commitment in SD, as is the case of Colombia, the case presented in this study. For this reasons, and given the importance of reaching a common ground to foster a regional dialogue that can enable a successful interaction with other regions of the world, especially to solve common global challenges, this paper addresses the very nature of SD through the eyes of the actors involved in the incipient Colombian SD initiatives, in order to contribute to the understanding of the intersection between STI and foreign policy and potential governance schemes in the southern world.

This study is developed under a phenomenological qualitative design and is also a case study. The phenomenological approach was chosen for this research, as it aims to understand the nature of SD as a phenomenon in emerging economies through the common experiences that different individuals have (Creswell, 2007), in order to develop (if any) a better governance at the intersection of STI and foreign policies in the southern world. The analysis was carried out through the inquiry of transcript semi-structured interviews conducted with actors involved in SD dynamics in Colombia to gain a better understanding of conceptions, practices, and suggestions for the future of SD.

In this paper, we present (i) a brief conceptual positioning regarding SD in emerging economies; (ii) the literature review tracking actors, practices, conceptions and suggestions, and a some key cases of SD in emerging economies; (iii) the general context of foreign and STI policy of the Colombian case and a vis-a-vis analysis of the international dimension of STI policy vs. the STI dimension of foreign policy in Colombia; (iv) the description of the undertaken methodology, this is the qualitative phenomenological case study approach for conducting analysis of SD in emerging economies; (v) the results of the analyses drawing from the SD actors, practices, conceptions and suggestions; (vi) a discussion around the type of governance and policy implications based on the actors' views and recommendations. Finally, (vii) the study concludes by suggesting the need of a policy mix for SD, proposing a potential general scheme and instrumentations for it in emerging economies. In addition, (viii) a set of practical recommendations and policy implications are proposed, and (ix) conclusions and further research questions are exposed, pointing out the importance of including non-conventional diplomacy actors and knowledge, and the need to inquire rationales behind possible SD policy mixes in the southern world.

The paper aims to contribute paving the way for emerging economies, and in particular for Latin American countries. The results show that SD actors in Colombia are scattered in a broad range of social sectors. From the government sector, academia, industry, civil society, individuals, international organizations, even indigenous communities, and NGOs. In terms of SD practices, the study recognizes that actor's actions are strongly related to the traditional diplomatic role of facilitating, bridging and connecting; and as a most frequent conception of SD, the participants consider, in line with the last point, that the main function of SD is “building bridges and connections.” Additionally, participants' suggestions about the promises of SD in emerging economies is related to capacity building, intermediation and the development of knowledge and skills, especially promoting scientists and other actors' training on SD. Besides, the research participants expressed the need to have a multi-stakeholder working group for such a purpose. This paper helps to better understand SD in a country that is in an early stage of converging STI development and foreign affairs. A governance scheme for SD in emerging economies is proposed as inspiration for potential policy mix instrumentation (Flanagan et al., 2011; Rogge and Reichardt, 2016).

## Conceptual Positioning

According to The Royal Society (2010) there are three dimensions for SD: (i) Science in diplomacy; (ii) Diplomacy for science; and (iii) Science for diplomacy. In general terms, they refer to the interplay between STI, international cooperation and policy. Flink and Schreiterer (2010) identify three goals at this interplay when countries devote efforts and resources to SD: countries are looking for (i) *Access* to resources (researchers, infrastructure/facilities, natural resources), (ii) the *promotion* of a country's achievements in R&D and other national assets, and (iii) *Influence* on the public opinion and decision-makers. In addition, and beyond the aspirational aim of governing sociotechnical systems for sustainability transitions (Smith and Stirling, 2006; Borrás and Edler, 2020), arguably nowadays, the globe is in the midst of an uncertain disruptive period that unavoidably implies a hard or smooth transition. In this context, SD could help to address global challenges derived from such a situation, and support the implementation of agendas tackling global as much as local priorities converging (Gluckman et al., 2017). This would be especially useful in late industrializing countries with high inequality rates (Rennkamp, 2011) as Colombia, which is characterized by less scientific human capital and technological resources that tend to be located in the Global North. Establishing meaningful alliances between governments such priorities that affect more vulnerable populations and ecosystems in these countries, can be addressed.

Having this in mind, the conceptual positioning of this paper, to propose a governance scheme for SD in emerging economies, lays out a policy mix approach. According to Flanagan et al. (2011) the common use of innovation policy mix notion can provide a reconsideration in how to better deal with complex, multi-level, multi-actor realities taking advantage of the interaction between policy instruments. Rogge and Reichardt (2013, 2016) giving one step beyond, recently stated that policy mixes refer to the combination of a policy strategy with multiple interacting instruments tackling a bunch of intimately related problems. Therefore, SD in and for emerging economies will be understood as a public arena where foreign and innovation policy instruments interact in order to address local challenges with a global scope, regularly ambitioning sustainability issues, given the disruptive context the world faces today. Furthermore, the policy mix notion framed by foreign affairs and STI issues may propose a complex governance scheme between these two domains, each time policy instruments have the power to structure the grammar of power and balances between governmental and non-governmental actors. In other words, that the potential instruments interacting in these two domains under “the umbrella” of a “unique” policy strategy are part of processes, dynamics, and designs of governance (Voß, 2007). Hence, SD, its “essence” and effects will be understood under the light of this perspective.

In general terms, SD can take many forms. However, more particularly in emerging economies, we consider it is featured by the conscious joint work of various actors, levels, mixes of policy instruments, and strategies in the interstice of foreign affairs and science, technology and innovation that can help to solve wicked problems derived from the current crisis that affect more southern countries, such as biodiversity loss or climate change.

## SD in Emerging Economies: Literature Review and Major Players

Among the literature of SD in emerging economies, case studies and experiences from Sub-Saharan Africa, Latin America, Russia, Serbia, among others are found. Most of the literature that delves into the concept of SD in emerging economies has a focus on specific instruments to promote STI cooperation (Hornsby and Parshotam, 2018), case studies focused on areas and dynamics of collaboration (Frech et al., 2018), capacity-building recommendations for individuals involved in SD (Krasnyak, 2020) and analysis of bi-regional agreements (Cherry and Du Toit, 2018). The literature on SD in emerging economies found to be relevant for the purpose of this study is summarized and presented in Table 1.

**Table 1:** References in literature of SD in emerging economies.

**Dimensions**	**Leverage points in literature of SD for emerging economies**
Actors	There are multiple references in the literature to non-traditional actors of diplomacy, also referred to as Track II Diplomacy. The actors of SD are diverse: Scientists, civil society, universities (Hornsby and Parshotam, 2018; Pantović and Michelini, 2018), as well as private entities, companies, and research centers (Ezekiel, 2020). Authors also mention hybrid collectives such as epistemic communities (Hornsby and Parshotam, 2018). However, actors belonging to the Track I Diplomacy or Official Diplomacy are also highlighted: Government officials, diplomatic corps and multilateral organizations (Ezekiel, 2020).
Practices	Practices mentioned in the literature refer mostly to the broader dynamics of governmental support to STI collaboration, however their purpose refers to the three goals proposed by Flink and Schreiterer (2010): Access, Promotion, and Influence. Practices include the establishment of alliances for international scientific cooperation, which can also support countries with tense relations (Patman and Davis, 2017). SD also refers to actions supported by governments to form international alliances for human development, the creation of facilities such as laboratories to support research, projects and platforms for inter-institutional collaboration and training of researchers, in order to achieve the interests of countries in STI (Ezekiel, 2020). SD practices include those that allow the insertion of developing countries in the knowledge economy and the management of resources for the development of solutions adapted to the contexts of emerging economies (Thompson, 2018). Fostering international alliances that bring countries from the Global North and South closer together and soften the relationship between countries with tense diplomatic relations (Patman and Davis, 2017).
Conceptions	When reviewing the dimension of conceptions, SD in emerging economies is mainly focused on *access*: development of capacities and exchange of resources in order to be part of the global scene of STI. For these countries, access to developments resulting from scientific research is not always available; also obtaining resources for their own developments that can support the search for solutions to diseases, problems and challenges is usually very difficult since these resources are not available (Thompson, 2018). SD also supports the economic development of emerging economies by inserting them into global value chains and mobilizing international experiences in order to build scientific and technical capacities of interest to the country (Hornsby and Parshotam, 2018).
Suggestions for the future of SD in developing countries	Suggestions for SD in emerging economies found in the literature include the need for scientists to assume leading roles in political debates, advising policy makers with scientific results and data (Patman and Davis, 2017). Foreign policy requires the integration of scientific evidence in their work to be able to support the implementation of SD initiatives (Ramírez-Cabrales and Rueda Forero, 2020). Suggestions also include the need to identify spaces for dialogue between academics, researchers and decision makers, in order to articulate projects to solve global issues and to address foreign policy priorities (Pantović and Michelini, 2018). Thompson (2018) argues that new models of SD, including diverse, non-traditional actors of diplomacy are needed to generate exchange and new knowledge.

In order to analyze the existing literature, four categories were determined, according to the research objectives of the present study: (i) actors, (ii) practices, (iii) conceptions, and (iv) suggestions for the future of SD in emerging economies, as these are cross-cutting categories that shed a light into the specific understanding and construction of the concept in the inquired context.

In addition, there are three major players that can depict the current trends of the concept in the Global South:

**Russia:** The development of SD in Russia was greatly impacted by the Cold War, which resulted in a “brain drain” phenomenon that reduced the number of researchers in the country, a considerable reduction of the government funding of STI activities, as well as the sanctions that were put in place after the accession of Crimea to Russia (Ibragimova and Barabanov, 2018). To the present, Russia recognizes the importance of SD and the need to integrate it within the country's foreign policy (Krasnyak, 2020). The most recent actions have been focused on restoring the international image of Russian research (Ruffini, 2017) developing a system of ongoing training and knowledge exchange in the field of scientific cooperation, in order to attract and retain Russian researchers and to provide better conditions for young professionals in STI fields (Ibragimova and Barabanov, 2018). The English-written literature does not mention any kind of governance structure or coordination among the multiple state actors that have a role in Russian SD such as the Federal Government, the Russian Academy of Sciences, the federal authorities, among others.
**India:** To promote SD, India highlights in its STI Policy the need to develop and achieve levels of global competitiveness through international collaborations, both bilateral as well as multilateral. This policy also reveals the importance of the acquisition, exchange and development of technologies through strategic alliances (Sikka, 2017). The main current approaches to SD in India are related to capacity building in science and technology, development of human talent, exchange and transfer of knowledge and the development of its institutions for science, technology and innovation. This is closely related to India's approach to supporting other developing countries, for example through SD initiatives such as scholarships awarded to foreign scientists and experts under the Indian Technical and Economic Cooperation Program (Arunachalam et al., 2017).
**Brazil:** As a major player in Latin America's Science, Technology, and Innovation landscape, Brazil has assumed a strategic approach as a leader within the South-South cooperation schemes. In this sense, the country's approach is to position itself as a powerful player in the Global South, becoming one of the strongest voices of emerging economies around the world and an important representation of the Latin American region. In Brazil, the concept of SD was first adopted and developed by the Ministry of External Affairs (Itamaraty), which decided to move away from the concept of SD to focus on Innovation Diplomacy, by creating the National Program on Innovation Diplomacy, thus putting a strong focus on the importance of STI in the socio-economic development and on Brazil's transformation toward a knowledge economy. In this sense, the national strategy of Innovation Diplomacy is focused on acquiring a new productive-technological profile to allow Brazil to redefine its international positioning and insertion in the global economy (Anunciato and dos Santos, 2020).

As it can be seen from the review above, the evolution of SD in emerging economies is very dependent on the specific historical and socio-economic conditions of each country or region; nevertheless, there are common leverage points among them as was presented in Table 1.

There is a gap and a need to discuss further the governance dimension of SD in emerging economies, particularly in the case of Latin America. The case of Brazil has been partially documented in scientific publications, mostly in Portuguese, but it is one of the few cases in which the governance of SD is analyzed by using a systemic approach (Anunciato and dos Santos, 2020). However, Brazil's Innovation Diplomacy national strategy has the characteristics of a top-down approach.

In Latin America, while many of the countries share similar historic and cultural traits, the evolution and integration of SD as a concept within the discourse of national STI and foreign policies is quite varied. Even though there is not enough academic literature that documents the cases of Mexico, Argentina, Chile, Cuba, Panamá, among others, a regional trend willing to promote and institutionalize SD is evident (Gual Soler, 2020). On the other hand, there are countries in the region that are still in the very early stages of defining a strategic approach and still struggle to gain the necessary momentum to reach a national agreement and commitment in this area, as is the case of Colombia, the case presented in this study. The UNESCO's policy brief on SD in Latin America and the Caribbean carried out by Gual Soler (2020) also refers to a set of challenges and opportunities from a regional perspective that are useful for thinking about the future of SD, especially in countries that are in the process of defining an initial roadmap. Among the challenges presented, the following are highlighted given their relevance for the present study: (1) The coordination and collaboration between institutions, actors, policies and functions at the intersection between science and foreign policy; (2) The fragmentation and multiplicity of high-level fora that currently exist; (3) The fluidity of the concept and the need to find a common ground; (3) The lack of institutionalization of SD, thus leaving actions disarticulated and without continuity; (4) The need to define the skills and knowledge required and promote capacity-building in this non-traditional field (Gual Soler, 2020).

Given the importance of reaching a common ground to foster a regional dialogue that can enable a successful interaction with other regions of the world, especially to solve common global challenges, there is a risk in having such a disparity in the evolution and understanding of the role of SD in Latin America. Therefore, while taking into account successful cases in different emerging economies, by studying the case of Colombia, this work intends to contribute paving the way for this country and other countries in the region that are also in the early stages of introducing the concept of SD within their science and foreign policy framework.

Moreover, the geopolitical relevance of Colombia due to its bi-oceanic condition, its global importance as a major source of the world's biodiversity and the fact of having put an end to one of the most devastating armed conflict of the western hemisphere in present times, makes it a case worth analyzing in the context of SD in emerging economies, which may contribute to tackling global challenges such as climate change or international security.

## The International Dimension of STI Policy and The STI Dimension of Foreign Policy in Colombia

On one hand, STI processes in Colombia have had a slow and interrupted development. Even today, the expected investment in STI activities has not been achieved, and the figures are not encouraging compared to other countries in the region. Colombia currently invests an average of 0.29% of GDP in R&D, which places it well below the average for OECD countries (2.35%) and also below the 0.73% average investment of Latin American countries (OECD, 2020). The international dimension of STI in Colombia indicates a low insertion of Colombian research in international scientific networks, given an absence of strong and long-term cooperation and coordination mechanisms and a lack of integrated actions to guarantee a strategic approach to STI internationalization that involves multiple stakeholders, including the scientific diaspora (Hernández et al., 2003; Misión de Sabios, 2019). According to the Cooperation Presidential Agency (APC), STI only mobilized 0,14% of international cooperation resources in 2019 and the country is still not playing an important role as an international cooperation provider in the South-South cooperation scheme (APC, 2019). These difficulties are the result of a lack of institutionalization and coordination to allow the development of strong international linkages and justify the need to define a SD strategy for the country (Misión de Sabios, 2019).

On the other hand, according to Amaya (2017) a historical analysis of the country's foreign policy has identified, among others, the following challenges: a predominance of short-term initiatives, a low capacity of the Ministry of Foreign Affairs to centralize the multiple dimensions of Colombia's international relations, institutional fragmentation, the rise of parallel diplomacies, discretion in the decision-making process, as well as a lack of spaces for debate with other actors. These institutional challenges, combined with the lack of prioritization of STI within the country's foreign relations agenda, result in the absence of a SD strategy in the country.

As in many other emerging economies, there are ongoing SD practices that have played an important role for the development of the STI system, especially through international cooperation initiatives promoted by governments and international organizations.

A series of isolated initiatives and examples (García, 2016; Bonilla, 2017) may prove that both Colciencias (now the Ministry of STI) and the Ministry of Foreign Affairs have contributed to support the internationalization of Colombian STI. Among those initiatives, the following two are some of the most illustrative examples of SD actions: (1) Red Caldas was a network of Colombian scientists, created and funded by COLCIENCIAS with occasional support from the Ministry of Foreign Affairs, to promote formal and informal linkages with Colombian researchers living abroad that functioned in the 1990s but failed to continue after the support from the government ended (Pellegrino, 2001); (2) the institutionalization of the honorary consuls through the 1538 decree of 2004, whose functions included, among others, the support of STI linkages with the host country. According to Isaza (2020), from this point on, high-level joint commissions have included delegates from different sectors, where some of the most important international agreements are reached. Their impact is, however, still difficult to grasp due to the challenges explained above.

The role played by Colciencias has been key for the implementation of SD initiatives in the country. For many years, the Head of the institution with the support of the internationalization unit of this Department represented the country in international high-level fora on STI, and developed specific financing instruments with matching funds negotiated through bilateral or multilateral agreements. However, constant changes in personnel and the lack of resources are a permanent concern in the organization (Plata, 2013). On the other hand, even though the country has led and been actively involved in several SD initiatives, there is no explicit intention from the Ministry of Foreign Affairs, to promote and support the design and implementation of a national SD strategy.

Nevertheless, what is described above, a series of recent milestones set now a proper scenario for a discussion on a more strategic approach to SD in the country:

1. The creation of the Ministry of STI in January 2019.
2. An official scientific mission called “Misión de Sabios” carried out in 2019. The report produced by the Mission includes a set of policy and instrument recommendations for strengthening the STI system in the long-term (Misión de Sabios, 2019).
3. The development of a new National Policy for Science, Technology and Innovation 2021–2030 expected to be launched in 2021, which integrates the recommendations from the Misión de Sabios report (Departamento Nacional de Planeación, 2020).
4. A diversification in Colombia's international agenda after the peace agreement was signed during the Presidency of Juan Manuel Santos. There is an evident search for a positive insertion through a high-profile presence in international fora, the return to multilateralism, the promotion of South-South cooperation, and the increasing role of paradiplomacy (Ardila and Clemente, 2019). The principles and guidelines of Colombia's Foreign Policy include the active promotion of a “diplomacy for sustainable development” and the support for other sectors to transform Colombia into an international attraction pole for education and STI (Ministerio de Relaciones Exteriores, 2018).

In this context, this analysis seeks to understand what kind of governance is needed and expected by the actors of SD in Colombia, by identifying and presenting conceptions, actors, practices and suggestions found at the intersection of science and foreign policy in Colombia. With this input, the aim is to suggest a series of elements that may contribute to a comprehensive policy and strategy on SD in the country for the advancement of STI and the country's sustainable development agenda, which may be adapted and extrapolated to other emerging economies.

In order to propose a SD governance scheme for Colombia, which may shed a light for other emerging economies; it is important to identify the essential elements at the intersection of STI and foreign policy. Thus, Table 2 presents the main elements found in policy documents that can be placed at this intersection, in order to complement the analysis of the findings and categorizations resulting from the interviewees' contributions. The aim is to provide a simple overview that could be used to enable a policy mix, a strategy intersecting foreign affairs and STI development, as well as a package of instruments with well-deliberated goals (Rogge and Reichardt, 2013, 2016).

**Table 2:** The international dimension in STI Policy and the STI dimension in Foreign Policy in Colombia.

**The international dimension in STI Policy**	**The STI dimension in Foreign Policy**
Statements from the Misión de Sabios (2019): • Through coordination and cooperation from the State, the strengthening and support of the scientific diaspora and the organized scientific networks of expatriates can become a platform to build trust between actors. • SD shall enable new paths for researchers and research processes from Colombia to the global research and innovation arena while contributing to the country's specificities and solving territorial needs. • The creation of specialized missions and diplomatic positions in several countries is recommended. Recommendations included in the National STI Policy (Departamento Nacional de Planeación, 2020): • Development of scientific-technical international cooperation agendas with counterparts to promote: Mobility of researchers, technology transfer, joint projects. • Increase the capacity of STI strategic intelligence and information to use scientific evidence for public policy. Development of a national prospective program with an emphasis in the 2030 Agenda. • Mobilization of international resources for STI. • Promote mission-oriented innovations. SD must be seen as a tool to implement global solutions and promote economic development and quality by inserting Colombia in international scenarios.	Statements from the Principles and Guidelines for Colombia's Foreign Policy 2018–2022 (Ministerio de Relaciones Exteriores, 2018): The 2022 vision presents Colombia as a leader through an innovative participation to provide answers to global challenges, and through actions to make Colombia a cultural, educational and tourism referent, as well as in matters of sustainability, entrepreneurship, and STI. • Development of an active “Sustainable Development Diplomacy” to achieve a better use of its natural resources, to protect and use its biodiversity and to tackle climate change effects (First action). • Foreign policy will contribute to the efforts of other sectors to transform Colombia into an international attraction pole for education, innovation, science and technology, boosting the country's capacities and generating incentives for the establishment of research centers and large joint projects. • A comprehensive, multi-dimensional policy of seas and oceans. • A comprehensive migratory policy and law that promotes labor, scientific and academic mobility (Fourth action).

In conclusion, the overview of STI and foreign policy in Colombia presented above indicates the need for a coordinated SD strategy. Moreover, the recent developments, milestones and explicit as well as implicit elements found at the intersection of these two systems as identified in the policy documents analyzed, are enough reasons to argue that the conditions are set for a more strategic approach to SD in Colombia.

## Methodology

### Research Objectives

With this research, we aim to understand from an exploratory perspective, the phenomenon of SD in emerging economies, taking into account the case of SD in Colombia, in order to propose a general governance scheme for SD in emerging economies at early stages regarding the topic. For this, we analyze the conceptions, practices, actors and suggestions for the future of SD in emerging economies, from the perspectives of SD actors in Colombia.

### Categories for Analysis

The categories of analysis were determined by prioritizing the importance of actors and actions (practices) for innovation policy mixes (Flanagan et al., 2011). The categories of conceptions and suggestions for the advancement of SD in emerging economies were taken into account, as these are cross-cutting themes that require further exploration.

### Design: Qualitative Phenomenological Case Study

This study has a phenomenological qualitative design and is also a case study. The phenomenological approach was chosen for this research, as it aims to understand the common experiences that different individuals involved in SD activities or initiatives have (Creswell, 2007) in order to develop a general model of a governance scheme pointing out the importance of a package of potential policy instruments at the intersection of foreign and STI policy. It is also a singular case study, as it seeks to investigate the phenomenon within a delimited context (Yin, 2003); in this case the Colombian experience of SD. According to Creswell (2007), the case study must identify a representative case, which can be used to generalize in similar contexts.

### Participants

The participants in this study were 18 actors involved in SD activities from the STI and diplomacy sectors in Colombia (see Appendix). Individuals coming from academia, government, scientific networks, associations, companies, and the Colombian scientific diaspora were interviewed. The participants were chosen, considering one or more of the following criteria:

- Years of experience in the fields of STI or diplomacy
- Knowledge of and interest in SD as an object of study
- Experience as part of SD initiatives or practices
- Representation of different regions of the country
- Representation of different sectors considered stakeholders of SD

### Data Collection

The data collection process was carried out during September 2020, through open-ended, semi-structured interviews. The interviews were conducted virtually and recorded with the consent of the participants. The interview guide had three dimensions related to the research objectives as follows:

- Dimension 1: Questions were asked about the actors' conceptions of SD, as well as about SD actors in Colombia.
- Dimension 2: Information was required on actions and practices carried out by the system actors related to SD.
- Dimension 3: The future outlook for SD in Colombia was explored.

### Data Analysis

Interview data were collected and recorded. The interviews were then transcribed. Under the methodology of qualitative content analysis and based on the information collected, inductive subcategories (codes) were created from the categories of analysis previously generated for this research. The creation of a taxonomy of categories, at the center of every content analysis, guarantees the addressing of the research question (Mayring, 2000). The qualitative analysis process was carried out with the support of the MAXQDA software, creating semantic networks.

The steps of the analysis process are described as follows:

- Codes were generated based on statements of interest for the objectives of research, using the MAXQDA software.
- After creating the codes, these were joined to the initial categories of analysis (conceptions, actors, practices and suggestions), generating semantic networks with MAXQDA.
- Since this is a qualitative study, quotations from the transcripts were chosen from the interviews, in order to present the results in a clearer way with examples.

## Results

The results are presented based on the four categories of analysis:, actors, practices, conceptions and suggestions for the future of SD in emerging countries.

### SD Actors

**Figure 1:**
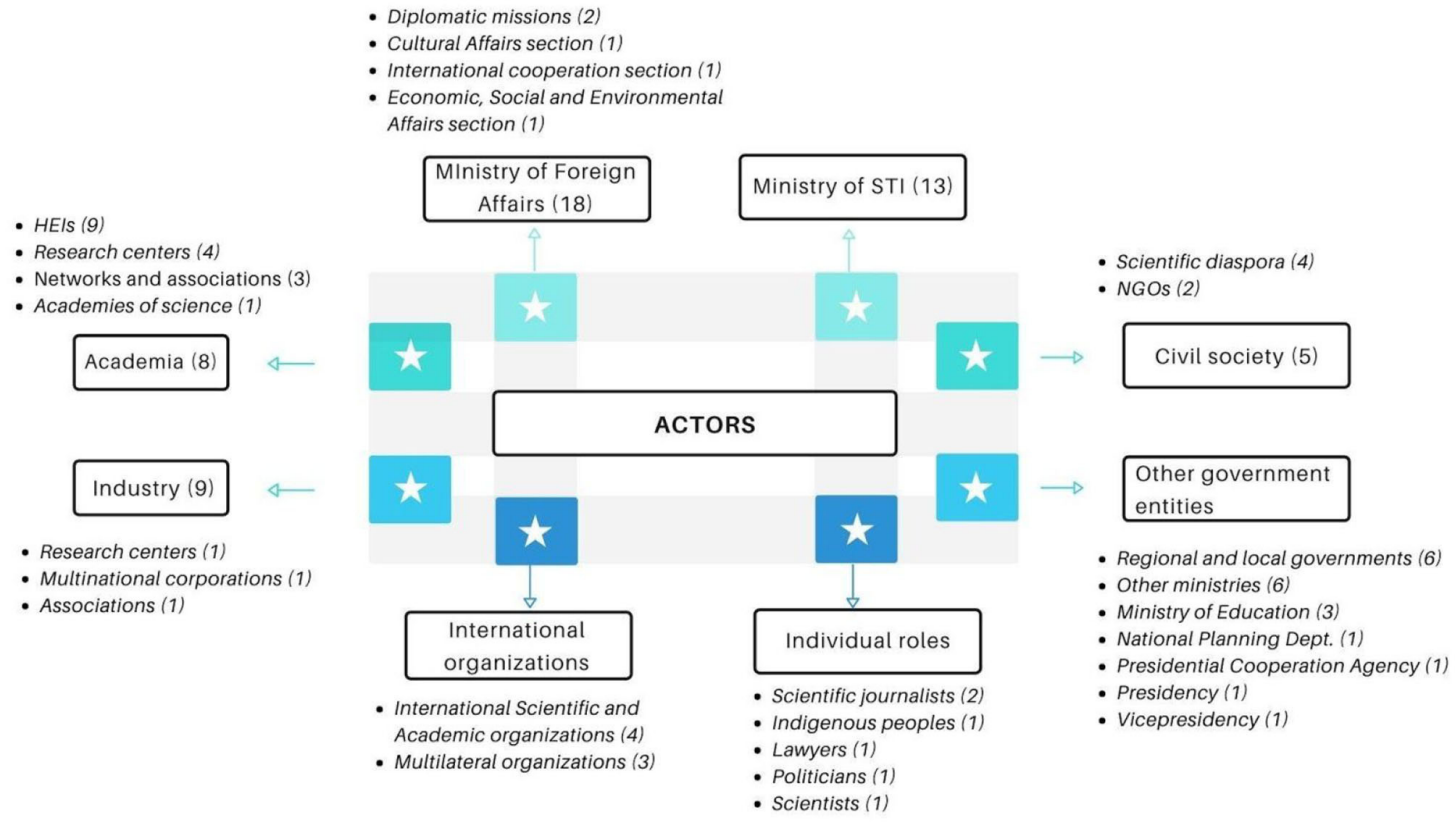
SD Actors in Colombia. Participants were asked to indicate the actors that should be considered within a SD strategy in Colombia. Categories are developed by the authors based on the type of actors (public, private, type of organization), as well as on frequency and similarity between the answers collected.

The SD actors (See Figure 1) range from the government sector, academia, industry, civil society, and individuals to international organizations. Among the government representatives, the Ministry of Foreign Affairs, through its diplomatic missions, and international cooperation, cultural affairs, and economic, social and environmental affairs stand out. The role of the Ministry of Science, Technology and Innovation is highlighted, as well as other government entities, such as the Ministry of Education and other ministries, the Presidency and Vice Presidency, the Department of National Planning and the Presidential Agency for Cooperation (APC). Interestingly, subnational authorities such as regional and local governments are also mentioned, a statement with strong relevance in centralized but culturally diverse countries like Colombia and other emerging economies.

Within the academic sector, interviewees mentioned higher education institutions, research centers, academic and scientific associations and networks, and academies of science. The industry was also indicated by multiple interviewees as a relevant actor, considering their role in promoting innovation and technology development activities. Among the industry, research and development centers attached to companies were mentioned and multinational corporations and industry associations. International organizations such as academic and scientific associations like the German Academic Exchange Service (DAAD), the German Research Foundation, Fulbright or the British Council were identified as actors but also as an important source for the definition of a strategy, given their role and the experience they have undergone in their own countries. Multilateral organizations, such as the UN or the OECD were also mentioned as relevant actors to promote SD initiatives in the country.

Many actors from the civil society were mentioned by the interviewees, such as NGOs, science journalists, indigenous communities, scientific diaspora, and other organized groups coming from multiple backgrounds and roles but gathered around a common interest. The latter were referred to as epistemic communities (Knorr-Cetina, 1981, 1999). For the case of emerging economies, especially in Latin America, both the scientific diaspora and the indigenous communities are two actors that should be further explored and integrated into the SD discourse of these countries. On the one hand, the interviewees indicated that the scientific diaspora should be at the center of a SD national strategy, given their ability to build international links and to understand different cultures. On the other hand, indigenous communities and the role of ancestral knowledge in SD aligns with the Government's effort, especially of the Ministry of STI, in giving a place and promoting all kinds of knowledge to acknowledge the diversity of the country and its multiple worldviews.

**Excerpts from interviews regarding the actors of SD in Colombia:**
– *National entities such as the Presidency, the Foreign Ministry, and, depending on the sector, should involve other ministries […] We also have the networks, the STI observatory, and the STI network attached to the Ministry of Science, Technology, and Innovation. In addition to sectorizing it, it is essential to lower it to regions. An international cooperation office was created in the Atlántico region. These offices must assume that role. The universities and multinational companies work with different countries and have the cultural knowledge and are interested in innovation (Participants 3)*.
– *SD is a cross-cutting theme: Higher education institutions should participate; from the government point of view: Foreign Ministry, Ministry of Science, Technology, and Innovation, the Presidential Agency for Cooperation (APC), Ministry of Education and research centers. At the regional level, the governorates, mayors and secretaries of education and STI, and the secretaries of economic development (Participant 5)*.
– *We have to recognize the value of our indigenous communities. Scientists are very interested in knowing and understanding the world of our indigenous peoples. Traditional, spiritual knowledge, the deep knowledge of nature, our pre-Hispanic knowledge. Indigenous peoples have their own form of diplomacy with the world outside their community (Participant 18)*.

### SD Practices

**Figure 2:**
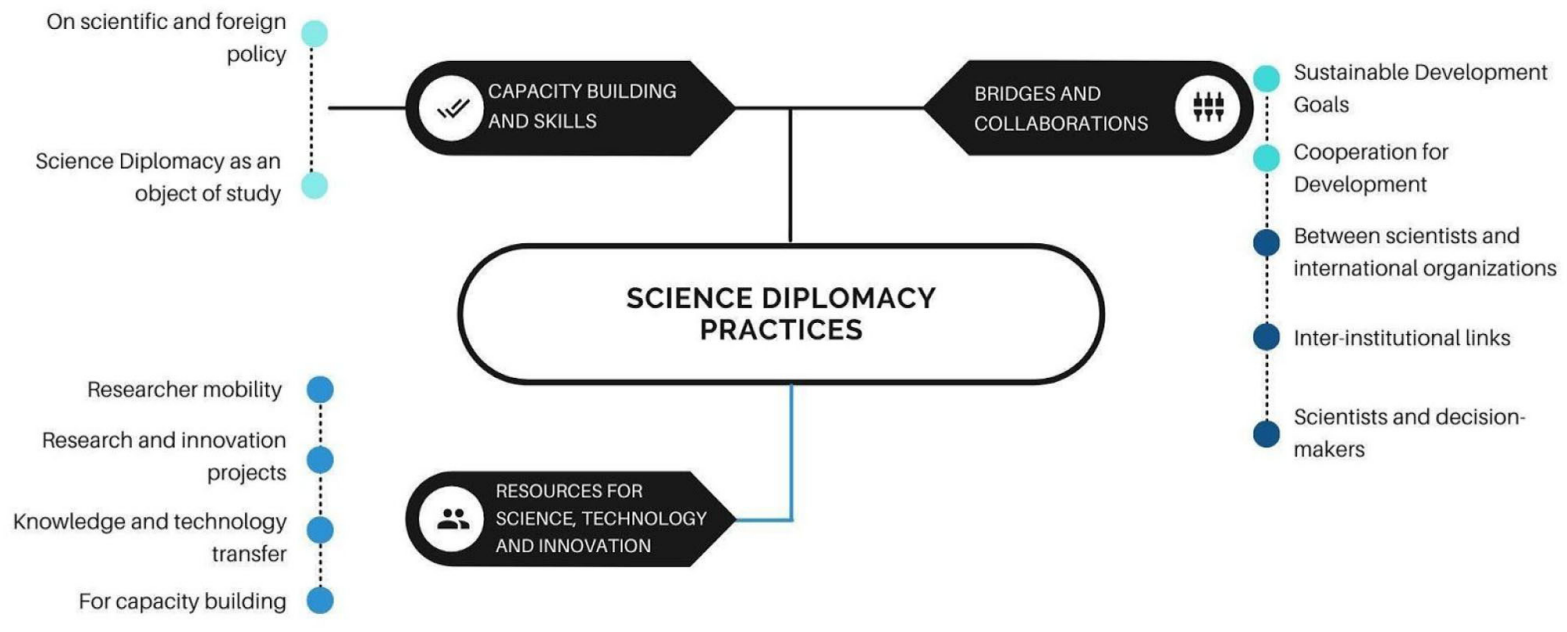
SD Practices in Colombia. Participants were asked to indicate, from their own specific roles or profiles, in what kind of practices associated with SD do they engage. Categories are developed by the researchers based on frequency and similarity between the answers collected.

The SD practices (See Figure 2) identified by the actors in Colombia are divided into three groups:

- Capacity building and skills,
- Resources for science, technology, and innovation,
- Bridges and collaborations.

In the category of capacity building and skills, participants identified the need to develop knowledge and skills for connecting science and foreign policy. In the second category called “Resources for STI,” practices such as the mobility of researchers abroad, research and innovation projects, knowledge and technology transfer and capacity building projects in STI are identified. Within the category “bridges and collaborations,” there are multiple sub-categories, including collaborations oriented toward the Sustainable Development Goals, cooperation for development, actions bringing together scientists with international organizations, generating inter-institutional links and connecting scientists with decision- and policy makers. In conclusion, for the interviewees, SD practices are strongly related to the traditional diplomatic role of facilitating, bridging and connecting.

**Excerpts from interviews regarding the practices of SD in Colombia:**
– *Something that we have called “Agenda setting”: Institutes like this can help carry out actions so that they can be considered for public policy, because they are science-based projects. I know more or less all the work of my researchers and they are really field researchers and for this reason Germany supports us because in Germany they cannot do field work on peace issues (Participant 7)*.
– *For research work, understanding that I can access international funds when we generate alliances with international researchers to access those resources (Participant 11)*.
– *From my work, from my research projects, I act as a bridge between academic communities in different countries and sometimes between decision makers (Participant 16)*.

### Conceptions on SD

**Figure 3:**
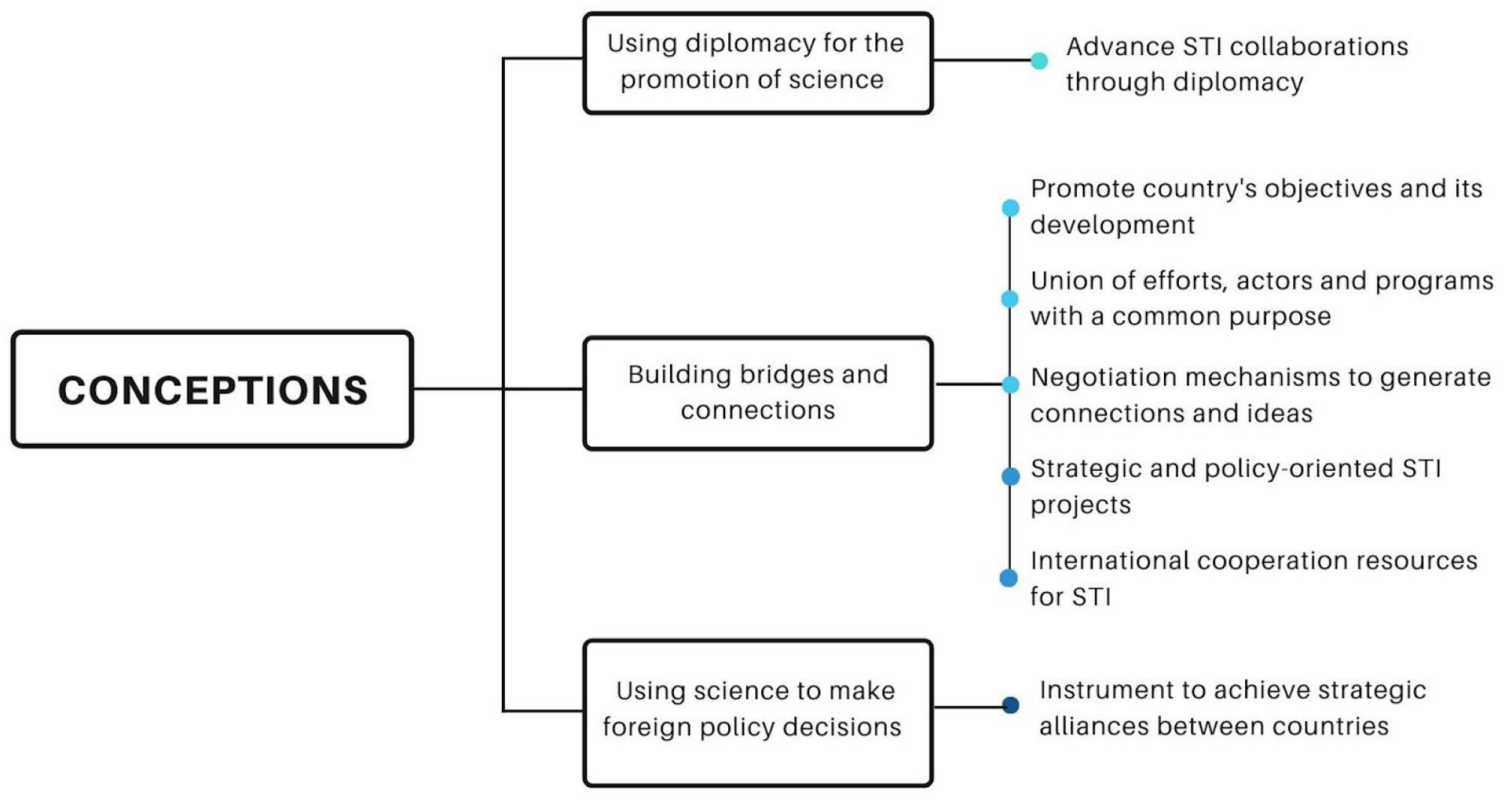
Conceptions on SD. Participants were asked to define SD. Categories are developed by the authors based on frequency and similarity between the answers collected.

The conceptions on SD by the actors are diverse (Figure 3); the most frequent conception among the participants is that SD refers to “building bridges and connections” for multiple purposes, namely, for international scientific cooperation for the solution of common challenges, for identifying strategic and political STI projects or for seeking international resources for STI. In this sense, SD is also seen as a mechanism to join forces with a common purpose, negotiate and generate connections for science or help a country achieve objectives with national interest. Other interviewees state more generally that SD refers to diplomacy to advance science, mainly supporting STI collaborations through diplomacy.

Finally, participants also commented that SD refers to science's use to make foreign policy decisions, being a strategic instrument to generate alliances between countries.

**Excerpts from interviews regarding the conception of SD in Colombia:**
– *I could say that SD is a union of efforts with a common purpose, between actors of the scientific community and of the country's foreign relations. It has a very important purpose, which is to achieve that link between scientific communities, global challenges, and how countries begin to work hand in hand with the scientific community to solve these global issues (Participant 4)*.
– *SD is a way to facilitate communication between two important aspects: the generation of knowledge through the scientific method and the need we have to maintain a good relationship with our neighbors, through good communication and sharing benefits for society (Participant 8)*.
– *SD facilitates, enables and accelerates the development of STI, through the instruments that are established, such as agreements (Participant 15)*.

### Suggestions for the Future of SD in Colombia

Capacity building, meaning the developing knowledge and skills for SD, was identified as the most relevant suggestion, especially promoting scientists and other actors' training on SD. Besides, the participants expressed the need to have a multi-stakeholder working group on SD. Some participants stated that the working group should be led by the Ministry of STI and the Ministry of Foreign Affairs in order to have enough traction to attract other actors. Another interesting suggestion had to do with aligning SD actions with the guidelines and recommendations provided by the *Misión de Sabios*. In a way, a natural match is evident between SD and the recommendations of the *Misión de Sabios*, as the international dimension included in the 2019 report explicitly refers to SD as a mechanism to advance toward a more robust STI system in Colombia and its impact in the development of the country's knowledge economy (Misión de Sabios, 2019).

**Figure 4:**
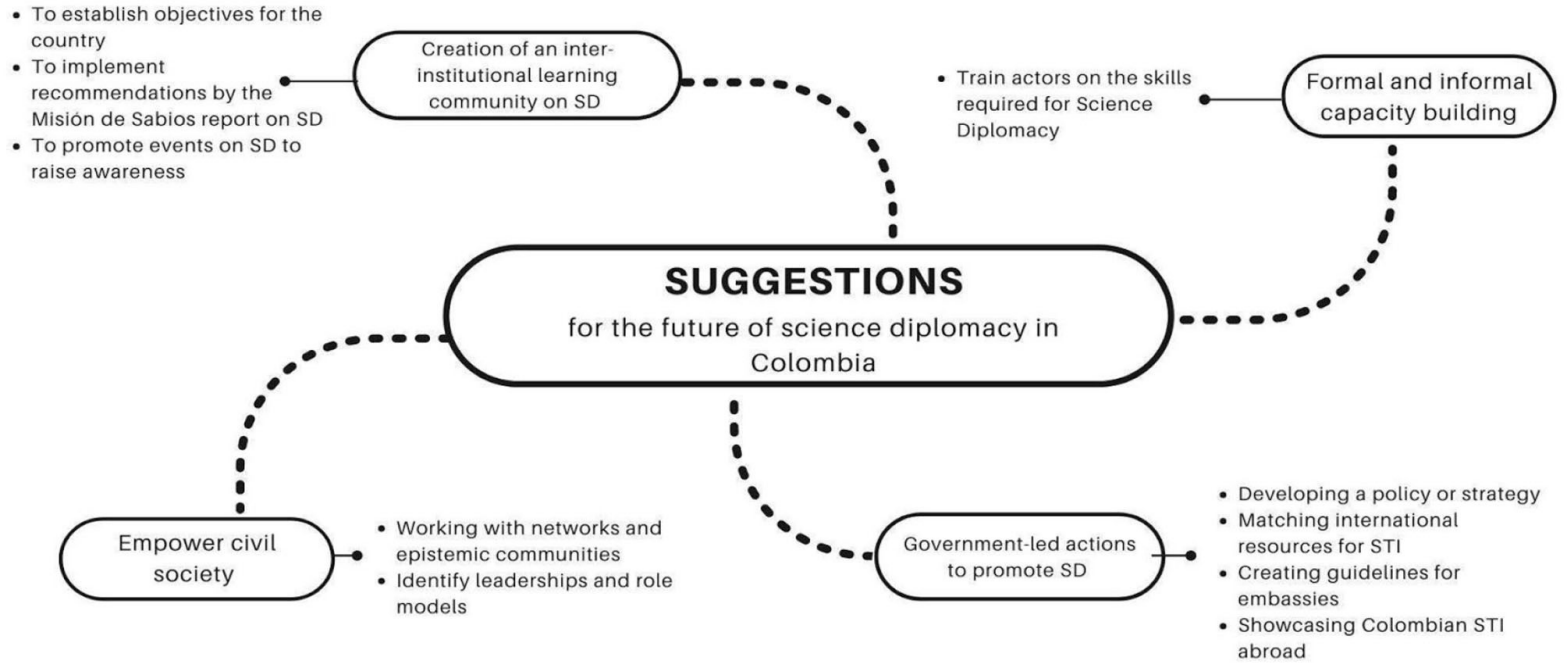
Suggestions to strengthen the integration of actors. Participants were asked to provide suggestions on how to advance SD in Colombia. Categories are developed by the authors based on frequency and similarity between the answers collected.

Likewise, the participants suggested official instruments to promote SD, such as the creation of a policy or strategy for the country. Some participants stated that such a policy should include clear guidelines for Colombian embassies to integrate SD activities in their agenda. An official instrument should focus on mobilizing international funds for STI, as well as promoting the Colombian STI production abroad. Finally, some participants also indicated that, given the multiplicity of actors that may play a role in this arena, civil society should be empowered by identifying leaders, champions and role models to inspire other actors. The role of networks and specifically multi-stakeholder groups like epistemic communities was highlighted as an excellent platform to advance toward an integrated strategy (Figure 4).

**Excerpts from interviews regarding suggestions to strengthen the integration of actors in Colombia:**
– *I have read the report from Misión de Sabios and there really are eight thematic axes with social and development impact. It is important that SD is articulated around these axes; In other words, all articulated efforts should be focused on these. One of the challenges of this report is to reach 2% of GDP in STI, through SD we can leverage resources to achieve this goal (Participant 1)*.
– *It is very difficult because there is no clear concept. One way to strengthen is to visualize cases and initiatives. When one reads literature on this, this is not a science from hypotheses, this is inductive, it is based on cases. Cases should be shown and studied so that people know them and tell the sectors that they have played important roles (Participant 7)*.
– *We need public and Government organizations to recognize and rely on organized civil society (Participant 16)*.

## Discussion

The conceptions of SD exposed by the interviewees are related to the construction of bridges and alliances with different objectives, especially for the development of international scientific cooperation, and to build capacities in STI, as stated by Hornsby and Parshotam (2018) for the development of solutions to global and common challenges (Ramírez-Cabrales and Rueda Forero, 2020). Among the conceptions, it is also observed that SD supports the achievement of objectives of national interest, promoting collaboration between countries with an impact on academic development and foreign policy decisions, as exposed by Pantović and Michelini (2018). There is also the conception of integrating science with national needs and foreign policy objectives, as stated by Krasnyak (2020) in the case of Russia. However, it is necessary to find meanings for SD in Colombia. In sister countries like Brazil, for example, there is a clear focus of its country strategy on innovation diplomacy, taking into account the interest on the part of this country to intersect in the knowledge economy (Anunciato and dos Santos, 2020). Gual Soler (2020) exposes the need to find narratives, conceptions and approaches for SD taking into account the Latin American context. In this sense, it is necessary to set building blocks for SD in the region and more particularly in Colombia.

When reviewing the actors of SD, the interviewees state that they range from the government sector, academia, industry, civil society and individuals to international organizations. In particular, the role of the Ministry of Foreign Affairs and the Ministry of Science, Technology and Innovation is observed, as well as other government entities of the national and regional order. Also other actors such as civil society, multilateral and international organizations and companies play a relevant role. Even entities such as universities and research centers are relevant actors in SD in emerging countries, as stated by Pantović and Michelini (2018). Non-traditional actors such as the diaspora, the indigenous and epistemic communities are gaining more relevance in the Colombian context and in emerging economies. This is articulated with what was stated by Thompson (2018), who explains the need to include non-traditional actors in national schemes of SD. As in the cases presented in the literature review, the inclusion of the scientific diaspora of emerging countries in SD schemes is imperative, especially for the execution of transnational scientific cooperation projects and to support the access to resources and experts.

On the other hand, SD practices in Colombia are divided into three areas: capacity building and skills development especially in STI, international resource management for STI and building bridges between the various actors of SD for projects and programs, oriented to the Sustainable Development Goals or global challenges, empowering and allowing a greater international visibility of emerging countries, as stated by Pantović and Michelini (2018). The development of human talent and research facilities are also a central practice of SD in emerging countries (Ezekiel, 2020). The focus of capacity building in STI, development of human talent and the exchange of knowledge and technologies was also observed in the case of India (Arunachalam et al., 2017). The use of SD to gain visibility and improve the image of the country through scientific production is one of the strategies also used by Russia (Ruffini, 2017).

Regarding the suggestions for the future of SD, capacity building must be further strengthened, especially by training scientists and other actors in SD skills adapted to the context of emerging economies. It is also necessary to create working groups where the different actors converge, in order to articulate the interests of the country and support the strengthening of science, technology and innovation systems, as stated by Pantović and Michelini (2018). The creation of a national strategy for SD is also required, which supports the mobilization of international resources for STI and includes the voices of the various actors, in order to promote the exchange and generation of new knowledge (Thompson, 2018). It is important to refer to the challenges identified by Gual Soler (2020), who states that Latin America must work to generate meaningful intersectoral collaborations for SD; institutionalize SD efforts, as well as generate learning roadmaps to develop the necessary skills in the region for SD among the involved actors.

## Policy Implications and A Tentative Governance Scheme of SD For Emerging Economies

Our research exposed the texture of SD in Colombia, a topic with a still varied and vague nature. However, specific conceptualizations regarding SD for emerging economies may be observed. According to the evidence, we can see that there are at least three rationales for SD expressed as conceptions:

- Building bridges for, on the one hand, cooperating with other countries toward solving common challenges, and on the other hand, to join forces internally to help the country solve issues of national interest with the support of science.
- Using diplomacy to promote the advance of national science.
- Using science to make or strengthen foreign policy decisions.

Arguably, these rationales support or may support the main practices identified:

- Collaboration practices for attaining sustainable development goals and for enabling multiple interactions: local-foreign scientists, exchanges between international organizations, between scientists and policy makers, among others.
- Capacity building.
- Mobilization of resources.

Finally, tentative futures expressed as suggestions for the improvement of the interaction and integration of actors in SD include:

- A. Capacity building on SD for researchers, policymakers among others.
- B. Creating a learning community for the development and promotion of SD.
- C. Empowering organized civil society. Having this in mind, we suggest the following governance scheme, as an example, for further analysis and discussion.

**Figure 5:**
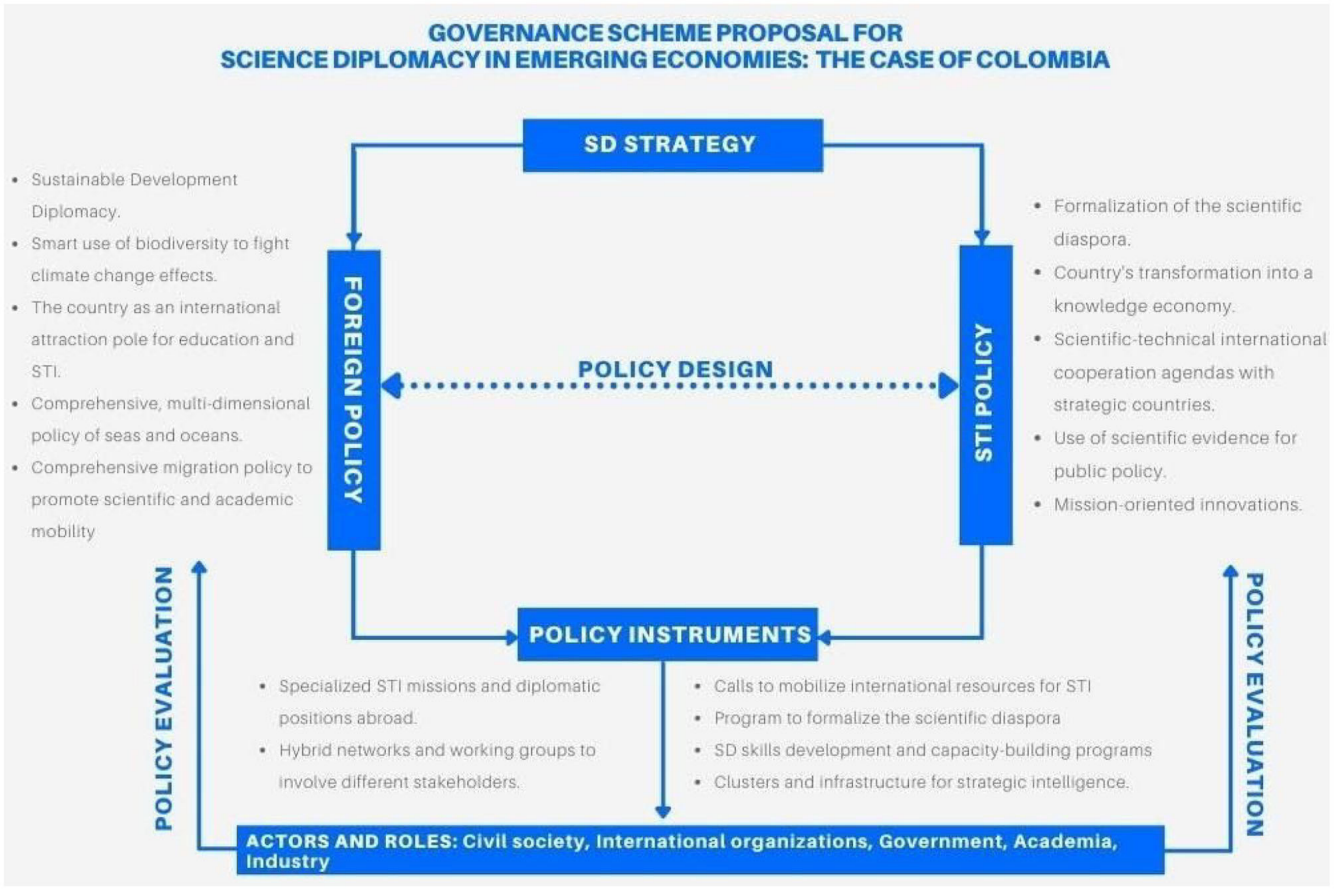
Governance scheme proposal for SD in emerging economies based on the Colombian case.

As is shown in Figure 5, an SD policy strategy is set for adjusting or making instruments that can distribute specific roles among and balance the power between the actors involved in SD in both administrative state sectors: STI and foreign affairs. The package of instruments designed and implemented could be tuned around leverage points that (i) gives a preponderant role to non-conventional actors (indigenous communities, NGOs etc.) referred in the analysis section above; (ii) facilitates bridging activities between these type of actors and scientist between countries (south-south-north) for attaining sustainable development ambitions; and (iii) offer *ad-hoc* guidelines for operationalize SD with this perspective in embassies worldwide. The potential instruments intersecting, must be evaluated in their implementation from novel policy evaluation tools, enough holistic to understand the impacts of these mixes. Policy learning and change would be possible if the conditions are accurate, which may result in the ulterior development of joint instruments for SD or specific ones that support the strategy.

This general overview would imply also a projection of how to proceed in terms of policy instrumentation which is not an easy task. The analysis presented in the precedent section can provide a general idea that inspires the analysis of bottlenecks and enable policy mixes intersecting foreign affairs and STI development aims (Rogge and Reichardt, 2013, 2016). Bringing together STI issues and foreign affairs under the light of a national development strategy foreseeing solutions of sustainable development in emerging economies, would help to understand how a governance of knowledge across borders can be deployed in late industrializing countries with high inequality rates (Rennkamp, 2011). Then, after considering overarching governance aspects that may support the articulation and coordination among actors, practices, and instruments between the two sectors, the following set of practical recommendations (see Table 3), is provided by gathering the suggestions from the local actors interviewed and those practices found in the literature and existing documented cases mentioned in previous sections. Recommendations are classified depending on the general SD practice they refer to, and the role each actor may play. Although some recommendations are ambitious, they can be further developed and serve as a tool to define the roles of each actor within SD, thus contributing to coordination and articulation.

**Table 3:** Set of recommendations for emerging economies in early stages of progress to advance SD schemes.

	**Actors**				
**Practices**	**Government**	**Academia**	**Industry**	**Civil society**	**International organizations**
Capacity building	Fund and participate in SD training in collaboration with academia Create a national intersectoral network or working group on SD Train diplomats and policymakers in SD	Generate a formal and informal academic offer of topics related to SD. Create doctoral and post-doctoral positions in SD Generate research groups on SD	Provide training on specific knowledge areas, such as the intersection of innovation and diplomacy Facilitate the transfer of new knowledge from industries abroad to the country for national interests	Provide training and organize events on SD topics, in order to bring society closer to schemes of SD Involve scientific diaspora in training activities as providers and beneficiaries	Advise governments on SD topics Open calls to develop projects and programs for Capacity building in SD between countries
Collaboration for SDGs/Global challenges	Create funding instruments for SD schemes Generate Mission-oriented and problem-solving research policies Participate in international negotiation scenarios Create networks and working groups with countries of the Global South to solve common problems	Tackle global challenges through the three missions: Teaching, Research, Outreach Generate specialized networks in order to provide knowledge and solutions Produce policy briefs to inform decision-makers	Provide knowledge and solutions through Corporate Social Responsibility Create action plans with clusters and associations to seek solutions to global problems	Transfer results of international collaborations to society Generate consultative committees for government entities, where diaspora participate. Introduce other kinds and sources of knowledge from indigenous communities, campesinos, informal workers, afro-descendant communities.	Generate spaces/events where scientists from different countries and government entities converge, to advance in the informed solution of problems
STI Resources mobilization	Generate bilateral programs with countries on issues of national, transnational or global interest Generate macro science-related events to make the research carried out in the country visible and attract researchers, investors and cooperation entities	Manage international resources within the framework of international scientific collaboration projects Create networking spaces between scientists from national and international universities and research institutions to apply for international calls and attract international resources	Manage resources with international companies for research and development projects that relate to national, transnational and global interests Support innovation diplomacy schemes for companies of all kinds Facilitate the transfer of technology of national interest to the country	Create incentives that promote international scientific collaboration for national, transnational and global interests Empower the organized diaspora by building specific work agendas for resource mobilization	Support the generation of SD schemes with transnational calls and funding for projects

*Based on the literature review and the interviews' results, a set of recommendations was developed crossing the categories generated for actors and practices of SD in Colombia, reformulating country-specific issues to enable a possible adaptation in similar contexts*.

This set of recommendations derived from our empirical analysis, beyond inspiring traditional instrumentation, such as international calls and programs to promote STI (diplomacy for science), leads to a reflection toward systemic policy instruments which promote and qualify interaction and strategic intelligence (Smits and Kuhlmann, 2004). This orientation applied to SD could reinforce or fulfill some of the still under construction Colombian innovation system functions and help to strengthen such a perspective in this country and beyond (Hekkert et al., 2007).

## Conclusions and Further Research Questions

Results show that SD in emerging countries such as Colombia could be oriented toward developing the STI systems through international scientific cooperation and scientific advice to advance public policy and foreign policy decisions. From interviewees' perspective, SD refers mainly to the generation of bridges and connections, especially related to the development of STI, for the search of solutions to global challenges, as well as national objectives. Likewise, the conception of SD articulated to scientific advice given to foreign policy decisions and actions was exposed. SD actors in Colombia range from the government sector, academia, industry, civil society, and individuals to international organizations. The role of universities is highlighted, as well as the importance of the scientific diaspora (Hernández et al., 2003), epistemic communities (Knorr-Cetina, 1981, 1999) and the need to include indigenous communities in SD schemes. Taking into account that other countries have advanced SD strategies articulating their academic and scientific diaspora, it remains pending how Colombia will execute a comprehensive strategy that can include the diversity of actors and knowledge, especially non-traditional actors of diplomacy.

SD practices evidenced in Colombia are related to what has been observed in other emerging economies analyzed in the literature, since there is an approach toward the development of capacities and skills, the management and access to resources for STI and the generation of alliances for projects, as well as foreign policy objectives. For SD schemes to be executed in the country, it is necessary to create a national strategy, taking into account national priorities and needs, as well *Misión de Sabios'* roadmap for the future of STI and the insertion of Colombia in the knowledge economy. More instruments to promote and stimulate international scientific collaboration are also required, as well as empowering the diversity of SD actors, including civil society. On the other hand, learning roadmaps should be developed to generate capacities and skills in SD for all the actors involved in Colombia, as well as learning communities to share cases and exchange experiences. The practical proposals set out in the Policy Implications section can support both Colombia and other emerging economies in the implementation of strategies that include the policy combination approach as a possible governance scheme for SD.

When thinking about global challenges, the analyses conducted under this academic piece show an evident lack of explicit directionality for SD. Moreover, only superficial references about the Sustainable Development Goals and barely any kind of missions are explained, despite the recent *Misión de Sabios in Colombia*. The actors interviewed make no explicit mentions to justice, peace, energy or post-extractive transitions (Gudynas, 2011; Andrade-Sastoque et al., 2020; Ordóñez-Matamoros, 2020), a very humble explicit mention about indigenous peoples, and no explicit references to the informal economy, campesino or afro-descendant communities and their importance as knowledge, research, and innovation actors in a multicultural country like Colombia (Andrade-Sastoque and Balanzó, 2017). In general terms, local urgent problems with high global impact and people related to them are conspicuous by its absence and the apparent apolitical dye of the participants' narratives. This can be further investigated.

From a phenomenological multi-stakeholder perspective, this study suggests that a governance scheme deserves to be discussed and designed to establish a long-term strategy that proposes a policy mix between STI policies and foreign policies to tackle very local issues with high global representativeness. Also the analyzed views from the actors interviewed, still show a limited scope regarding rationales, activities and proposals to improve actor interaction for SD, in two senses: (i) the specific aims when referring to sustainable development and global challenges, and (ii) local urgent problems with high global impact.

Despite the potential of the SD governance general scheme for emerging economies presented and suggestions for policy instrumentation, still questions remain to be further investigated:

– *What rationales may be more accurate for SD in emerging economies?* Studies should draw on the current literature on SD and identify empty spaces regarding the specificities identified in emerging economies, especially regarding STI systems and dynamics. This may pave a way to enrich the SD discourse and develop more valid rationales for emerging economies.
– *How does SD practice contribute to solving global challenges without neglecting profound local problems?* An in-depth analysis of the logics behind the Sustainable Development Goals discourse in Colombia should be carried out, considering the analysis and recommendations from the *Misión de Sabios*, to focus the efforts and avoid neglecting local problems. Issues such as peace, energy or post-extractive transitions deserve further study.
– *Who can be new entrants or invisible actors involved in SD in emerging economies?* As explained above, actors such as indigenous peoples, informal economy workers, *campesino* or afro-descendant communities and their relevance as knowledge, research and innovation actors in a multicultural country like Colombia is a subject that deserves further analysis in the framework of SD studies.

Finally, we propose advancing in developing a specific narrative of SD in emerging economies like the Colombian one, which considers their specific context, conditions, needs and motivations. The interplay between rationales, practices and futures should be included in a SD policy that tackles local problems globally.

## Data Availability Statement

The datasets presented in this study can be found in online repositories. The names of the repository/repositories and accession number(s) can be found below: https://cutt.ly/ojnrRET.

## Author Contributions

All authors listed have made a substantial, direct and intellectual contribution to the work, and approved it for publication.

## Conflict of Interest

The authors declare that the research was conducted in the absence of any commercial or financial relationships that could be construed as a potential conflict of interest.

## Appendix

**Table A1:** Research participants and profiles.

**Participant (P)**	**Profile**
P1	Director from an International Office at a Colombian university.
P2	Director from a Research Institute.
P3	STI Independent Consultant.
P4	Vice-chancellor for research at a Colombian university.
P5	Head of International Relations from a governmental entity.
P6	Head of International Cooperation from a network of Colombian universities.
P7	Administrative Director from a Research Institute.
P8	A representative from the Pharmaceutical Industry.
P9	Colombian scientist and entrepreneur living in Brussels; has a company dedicated to promoting RandI collaboration between Latin American universities and Europe.
P10	Director from one of the sections at the Ministry of Foreign Affairs.
P11	Researcher at a Colombian University. Experience working at a US institution and with knowledge of public policy guidelines.
P12	Researcher at a Colombian University. Experience as a researcher in Europe and maintains close collaboration with European partners. Director of a doctoral program.
P13	Colombian scientist and entrepreneur living in Berlin. Designated a Research and Innovation ambassador for the city of Berlin. A company dedicated to promoting EU-LATAM links between academia and companies, mainly in the biotech sector.
P14	Director of the Diplomatic Academy of the Ministry of Foreign Affairs.
P15	Manager of RandI incentives at a large dairy company.
P16	Researcher at a Colombian University. Part of an European project focused on promoting and studying SD in Europe.
P17	The executive director of a Scientific Association. Currently doing research on SD.
P18	Former Minister of Environmental Affairs, former Ambassador of Colombia in Germany. Member of multiple national and international boards on global issues.
